# Nanoencapsulation Transforms Curcumin’s Antiparasitic Activity: Reduced Dose and Altered Mechanism in *Taenia crassiceps* Cysticerci

**DOI:** 10.3390/antiox15101226

**Published:** 2026-09-23

**Authors:** José de Jesús Martínez-González, Lucero Reyes-García, Alberto Guevara-Flores, Martín Escamilla-del-Arenal, Rachel Austin Duffié, Gabriela Nava-Balderas, Gerardo Leyva-Gómez, María Luisa Del Prado-Audelo, Irene Patricia del Arenal Mena

**Affiliations:** 1 Departamento de Bioquímica, Facultad de Medicina, Universidad Nacional Autónoma de México (UNAM), Mexico City 04510, Mexico; jjmtz@facmed.unam.mx (J.d.J.M.-G.); qfb.reyes.garcia@gmail.com (L.R.-G.); guevarafa@comunidad.unam.mx (A.G.-F.); 2 Department of Biochemistry and Molecular Biophysics, Mortimer B. Zuckerman Mind, Brain, and Behavior Institute, Columbia University, Jerome L. Greene Science Center, 3227 Broadway, New York, NY 10027, USA; martin.escamilla13@gmail.com; 3 Instituto de Ecología, Universidad Nacional Autónoma de México, 04510 (UNAM), Mexico City 04510, Mexico; rachel.duffie@gmail.com; 4 Departamento de Microbiología y Parasitología, Facultad de Medicina, Universidad Nacional Autónoma de México (UNAM), Mexico City 04510, Mexico; gabrielanavab@yahoo.com.mx; 5 Departamento de Farmacia, Facultad de Química, Universidad Nacional Autónoma de México (UNAM), Mexico City 04510, Mexico; leyva@quimica.unam.mx; 6 Escuela de Ingenieria y Ciencias, Tecnológico de Monterrey Campus Ciudad de Mexico, C. Puente 222, Mexico City 14380, Mexico; luisa.delprado@tec.mx

**Keywords:** curcumin, *Taenia crassiceps*, nanoparticles, lethal dose, nucleolus

## Abstract

Free curcumin has been shown to have a lethal effect on *Taenia crassiceps* cysticerci, due to its ability to interfere with the parasite’s redox metabolism. Free curcumin is highly unstable, which limits its bioavailability and reduces its efficacy as an anthelmintic drug. To overcome these constraints, we evaluated the use of Polycaprolactone-Pluronic©-F68 nanoparticles (PCL/F68 NPs) to improve curcumin stability for in vitro administration against cysticerci. Our results indicate that PCL/F68 NPs effectively protect curcumin in an aqueous medium, and after 120 h incubation with 10 µM curcumin-loaded NPs, cysticerci mortality was 100%. In contrast to free curcumin, whose cysticidal activity is associated with increased ROS production, disruption of the parasitic thioredoxin antioxidant system, and tegumental damage, curcumin-loaded NPs induced cysticerci mortality in the absence of these effects and were instead associated with profound nucleolar alterations, implicating nucleolar dysfunction as a potential mediator of parasite death. The reduced dose, increased bioavailability and enhanced antiparasitic efficacy suggest that PCL/F68-CUR NPs offer a promising therapeutic strategy for treatment of cysticercosis.

## 1. Introduction

Neurocysticercosis, a parasitic infection caused by *Taenia solium* (order Cestoda) is recognized by the World Health Organization (WHO) as one of the neglected tropical diseases, affecting populations in Latin America, South and Central Africa, and Southeast Asia [1]. When this parasitic flatworm lodges in the nervous system (neurocysticercosis), severe epilepsy can result [2]. Because cysticercosis is associated with significant health and socioeconomic burdens [3,4], the search for new, affordable pharmaceuticals is urgent.

Curcumin, a curcuminoid obtained from the rhizome of *Curcuma longa* has been found to interact with multiple molecular targets, consistent with its pleiotropic biological effects and its potential application in the treatment of various chronic diseases [5]. As an antiparasitic, curcumin has recently gained attention for its ability to interfere with key physiological processes in parasites [6,7]. In cestodes, the thioredoxin antioxidant system represents a particularly attractive target, as thioredoxin glutathione reductase (TGR) [8] differs from that of the host (TrxR). Supporting the therapeutic potential of this pathway, we previously demonstrated that both auranofin [9] and curcumin [10,11] inhibit cestode TGR activity. Furthermore, free curcumin exerts a dose- and time-dependent lethal effect on *Taenia crassiceps* cysticerci in vitro. This effect is attributed partly to the inhibition of the TGR, which promotes the accumulation of reactive oxygen species (ROS), and ultimately parasite death due to oxidative stress [11].

Despite its wide range of biological activities, important pharmacological limitations have hindered curcumin-based therapeutic advances [6]. Curcumin exhibits extremely low aqueous solubility, poor chemical stability at physiological pH, rapid degradation through autooxidation, and limited bioavailability due to fast metabolism and clearance [12,13]. A major contributor to its instability is its propensity to undergo rapid autooxidation in aqueous environments, a process described in detail by Schneider et al. [12]. During this transformation, curcumin is readily converted to reactive intermediates [14], which can reduce its effective concentration and compromise reproducibility of biological responses. Collectively, these constraints make direct pharmaceutical administration of free curcumin challenging, particularly in systems where sustained exposure is required.

Consequently, strategies capable of protecting the molecule, improving its dispersion in biological media, and modulating its release profile are necessary to fully exploit its therapeutic potential. In this context, nanoformulation-based delivery systems have emerged as a promising approach to overcome these limitations and enhance the biological performance of curcumin. Nano disks [15,16], curcumin-loaded lipid-core nanocapsules (C-LNCs) [17], and Cur- Bio PLGA NPs [18] have been used to this end. We previously developed curcumin-loaded Polycaprolactone/Pluronic^®^ F68 nanoparticles (PCL/F68 NPs) which proved to be suitable for solubilizing and protecting this molecule and have shown good biocompatibility with neuronal and glial cells [19], in addition to exhibiting cryoprotective and antioxidant properties [18,20]. Although several nanoformulations of curcumin have been reported, little is known about whether encapsulation modifies the mechanism of action of curcumin against parasitic helminths [21].

The objective of this study was to evaluate the efficacy of a nanoformulation designed to stabilize curcumin, thus facilitating its pharmaceutical administration to cysticerci. We use the research model *Taenia crassiceps*, a non-infectious cestode to circumvent challenges of working with *T. solium*. *T. crassiceps* reproduce asexually by budding when inoculated in the mouse peritoneum, which ensures a continuous supply of larval-stage cysticerci [22,23]. Our results demonstrate that PCL/F68 nanoparticles enable the administration of curcumin in vitro and are associated with marked nucleolar alterations without the consistent TGR inhibition and ROS increase response previously observed with free curcumin. These findings suggest that NP-CUR may induce *T. crassiceps* cysticercus mortality through a cellular pathway distinct from that previously described for free curcumin.

## 2. Materials and Methods

### 2.1. Chemicals and Reagents

Poloxamer 188 (Pluronic^®^-68, P68; catalog number 15759), polycaprolactone (PCL, catalog number 440752) and DMSO were purchased from Sigma-Aldrich^®^ (Merck, Darmstadt, Germany). RPMI-1640 medium was obtained from Life Technologies-Gibco (Grand Island, NY, USA). 5-(6-) chloromethyl-2′,7′-dichlorodihydrofluorescein diacetate (CM-H_2_DCFDA) was obtained from Thermo Fisher Scientific (Eugene, OR, USA). Curcumin was acquired from Toronto Research Chemicals Inc., (North York, ON, Canada). All other chemicals were purchased from Sigma-Aldrich.

### 2.2. Biological Material

Ten 8-week-old female BALB/c mice were inoculated intraperitoneally with 15cysticerci of the *T. crassiceps* HYG strain. The animals were divided into two cages and placed in a room under a 12 h light/dark cycle and constant temperature (21 ± 2 °C) and allowed libitum access to a standard laboratory diet and purified potable water. The parasite’s proliferation was monitored by checking the weight gain of the infected mouse as well as the increase in the mouse’s abdominal volume. At the same time, the health of the mice was monitored by regular visual inspection, using the parameters evaluated by the mouse Grimace scale (https://nc3rs.org.uk/3rs-resource-library/grimace-scales/grimace-scale-mouse (accessed on 18 May 2023)). Three months later, mice were euthanized by cervical dislocation, and cysticerci were recovered from the peritoneal cavity under sterile conditions. Before use, larvae were washed thoroughly with sterile 10 mM phosphate-buffered saline solution (PBS) at pH 7.4 [23] and selected manually to obtain a cysticercus population of uniform size (3.0 mm diameter) by using a sterile metal sieve. The conditions for cysticercus maintenance in culture medium have been previously described [24]. Briefly, 100 cysticerci (approximately 2 mL) were carried to 20 mL with RPMI-1640 medium (pH 7.0) supplemented with 1% penicillin streptomycin. After the treatment, cysticerci were recovered, washed with PBS and separated into aliquots to measure viability, TGR activity and ultrastructure.

To test the effect of PCL/F68-CUR NPs (NP-CUR) on *T. crassiceps* cysticerci under culture conditions, cysticerci were exposed to different equivalent concentrations of CUR (2, 5 and 10 μM; final concentration in culture medium) for different periods of time (24, 48, 72, 96 and 120 h). Untreated cysticerci and cysticerci exposed to PCL/F68 NPs (empty NPs) were used as controls.

#### Ethical Statement

All animal procedures were conducted in strict accordance with the ethical guidelines for the care and use of laboratory animals and were approved by the Institutional Animal Care and Use Internal Committee (CICUAL) of Facultad de Medicina, Universidad Nacional Autónoma de México (FACMED-UNAM) under protocol number 008-CIC-2023 (Project approval 18 May 2023, to 6 June 2027). The study adhered to the national guidelines of the NOM-062-ZOO-1999 and procedures were in accordance with the recommendations of the U.S. National Research Council’s Guide for the Care and Use of Laboratory Animals. All efforts were made to minimize animal suffering, maximize animal welfare and reduce the number of animals used.

### 2.3. Nanoparticle Preparation and Physicochemical Characterization

Nanoparticles (NPs) of the biopolymer polycaprolactone (PCL), together with the surfactant Pluronic^®^ F68, were loaded with 50 mg curcumin (NP-CUR) or without curcumin (empty NP), as previously reported [19]. The determination of size, polydispersity index (PDI) and zeta potential was carried out using Zetasizer Nano ZS90 (Malvern Panalytical Ltd., Malvern, UK). Transmission electron microscopy (TEM) was used to evaluate the shape of the generated NPs. For this purpose, a drop of the nanoparticle suspension was placed on parafilm, and a copper grid with Formvar/Carbon was placed on top for approximately 20 min. Subsequently, the drop was removed, the excess was allowed to dry, and the sample was placed in another drop of aqueous uranyl for 10 min. After two washes, the sample was observed with a JEM-1010 Transmission Electron Microscope (JEOL Peabody, MA, USA) equipped with a Hamamatsu camera (Hamamatsu Photonics, Japan).

#### Quantitation of Curcumin in NP-CUR

A curcumin standard curve with 5 to 30 μM prepared in DMSO was generated (correlation coefficient of R^2^ = 0.99985) and absorbance was detected by spectrophotometry at 426 nm (Life Science UV/Vis Spectrophotometer, DU^®^730, Beckman Coulter, Stanwood, WA, USA). To calculate the amount of curcumin loaded in NP-CURs, 40 μL of freshly prepared NP-CUR suspension was dissolved in 500 μL DMSO, mixed and later centrifuged at 14,500× *g* for 30 min at 4 °C. The supernatant was recovered and the amount of released curcumin was calculated by extrapolating the absorbance at 426 nm using a calibration curve after subtracting the absorption of an equivalent volume of empty NP suspension.

### 2.4. Evaluation of the Effect of Nanoparticles on Taenia crassiceps Cysticerci

#### 2.4.1. Exposure of Cysticerci to Nanoparticles Under Maintenance Culture Conditions

The effect of NP-CUR in *T. crassiceps* cysticerci under culture conditions was tested. To this end, cysticerci were exposed to different equivalent concentrations of curcumin (2, 5 and 10 μM; final concentration in culture medium) for different periods of time (24, 48, 72, 96 and 120 h). Untreated cysticerci and cysticerci exposed to empty NPs were used as controls. Once the incubation period was complete, the cysticerci were recovered, washed three times with cold PBS, and divided into aliquots to evaluate the effect of NP.

After cysticerci were incubated with the NP-CUR at the concentrations and time described above, in addition to the images shown, the following parameters were measured in accordance with the methodology previously reported [25]: percent viability, TGR activity, and quantification of oxygen reactive species (ROS).

#### 2.4.2. Cysticercus Viability

Cysticercus viability was evaluated in aliquots of 50 cysticerci by both vital staining (0.02% trypan blue) and temperature-induced motility, as previously reported [24].

#### 2.4.3. TGR Activity in Crude Extract

Aliquots of 50 cysticerci were used to determine enzyme activity for each sample (controls and treatments, respectively). Each aliquot was placed in a 1.5 mL Eppendorf tube and kept at 4 °C. Subsequently, 86 μM PMSF was added, and cysticerci were homogenized with a manual tissue homogenizer (Bel Art, SP Scienceware, Wayne, NJ, USA). The homogenates were centrifuged at 14,000 rpm in a microfuge Hermle Z 180 M (Labnet International, Edison, NJ, USA) for 1 h at 4 °C. Supernatants were recovered and dialyzed using a 3500 Da molecular weight cut-off (MWCO) membrane (Spectrum, Thomas Scientific, New Brunswick, NJ, USA) against 20 volumes of Tris 100 mM EDTA 1 mM pH 7.8, with two buffer changes.

Once the samples were dialyzed, the disulfide-glutathione reductase activity of the TGR was assayed based on the NADPH-dependent reduction of the substrate GSSG, which is monitored by the decrease in absorbance at 340 nm, as described previously [24]. To calculate the specific activity of the enzyme, the protein concentration was determined using the Markwell method [26].

#### 2.4.4. Quantification of Total-ROS by Fluorescence

The redox dye CM-H_2_DCFDA was used to determine ROS in whole cysticerci as we described previously [11]. Aliquots of 10 cysticerci from each control and treatment sample were collected in 0.5 mL graduated Eppendorf tubes and brought to a total volume of 300 µL with PBS 1X in darkness. Ten µL of 300 µM CM-H_2_DCFDA stock solution was added to each tube and vortexed. To facilitate the entry of the fluorescent probe into the cysticerci, the samples were incubated for 30 min with gentle agitation (126 rpm) at 37 °C. Later, cysticerci were recovered, washed with cold PBS and placed in a 96-well plate. The fluorescence of oxidized dye was recorded using an excitation/emission wavelength of 485/535 nm, respectively. The results were normalized based on the baseline production of ROS presented by untreated cysticerci.

### 2.5. Processing of Treated Cysticerci for Microscopy Techniques

#### 2.5.1. Curcumin-Loaded NP and Its Localization on Cysticerci by Fluorescence Confocal Microscopy

For the observation of cysticerci exposed to NP-CUR for 1 and 120 h, samples of one rinsed specimen were placed in a slide and covered with a coverslip to immobilize live cysticerci (sample of 1 h). Fluorescence imaging of NP-CUR into cysticerci was observed using a fluorescence confocal microscopy ZEISS LSM 880 (Carl Zeiss, Oberkochen, Baden-Wurtemberg, Germany).

#### 2.5.2. Analysis of the Ultrastructure of Cysticerci Exposed to NP-CUR by TEM

Samples of untreated cysticerci (control), and those exposed 120 h to empty NPs and NP-CUR (10 µM), were fixed in Karnofsky solution and processed according to the method reported by Valdespino-Vázquez et al. [27]. The semi-thin sections (1 µm) were stained with toluidine blue and thin sections (120 nm) of the embedded sample were obtained in Leica EM UC7 ultramicrotome and were placed on copper grids. The samples were observed in a Scanning Transmission Electron Microscope (Crossbeam 550; Carl Zeiss, Oberkochen, Baden-Wurtemberg, Germany) at 10 kV.

#### 2.5.3. Electron Microscopy Analysis of Nuclei

Due to the complex structure of the cysticercus, the presence of a double membrane was the criterion used to confirm the identification of nucleus. Each nucleus TEM image was analyzed separately to determine the presence of a nucleolus and its integrity. Results were plotted using R software (Version 4.5.2., R Foundation for Statistical Computing, Vienna, Austria). Significance was determined by the Z test for two proportions, from a total number of 95 images (control = 28, empty NP = 23 and NP-CUR = 43) from W distinct organisms (control = X, empty NP = Y, and NP-CUR = Z).

#### 2.5.4. Gray Intensity Estimation

All analyses for images acquired after electron microscopy were completed in FIJI and R studio. Average Signal Intensity: A line was traced with the Straight Segment tool through the nuclei. The resulting intensity profile was generated using the Plot Profile function, and gray intensity values were extracted along the length of the line. To allow comparison between images, gray intensity values were normalized within each image, and the distance across the nucleus was normalized such that the beginning and end of the nuclear measurement corresponded to 0% and 100%, respectively. Normalized intensity profiles were analyzed in R software (Version 4.5.2., R Foundation for Statistical Computing, Vienna, Austria). Statistical comparisons were performed using Fisher’s Z test on the normalized, unsmoothed intensity data. For visualization, intensity profiles were represented using a locally estimated scatterplot smoothing (LOESS) curve. Google AI Gemini Version 3.5 was used to debug the R-code that was used to estimate *p*-value.

### 2.6. Statistical Analysis

Results are expressed as mean ± standard derivation (SD) or standard error of the mean (SEM). Activity experiments were performed in triplicate and analyzed using a one-way ANOVA test and Tukey’s post hoc test to evaluate the effect of NP-CUR on cysticercus viability, TGR activity and ROS production, for each of the time periods evaluated. In all cases, the data were normalized relative to the corresponding time-matched control. TEM images were quantified using a multinomial mixed-effects logistic regression model and Fisher’s Z tests. Statistical analysis and graphing were performed using OriginPro software (Version 9, OriginLab Corporation, Northampton, MA, USA). *p*-values of <0.05 were considered statistically significant.

## 3. Results

### 3.1. Characterization of the PCL/F68-CUR NPs

The nanoparticle (NP) prepared for this work was made using the emulsion method. Physicochemical characterization of the NPs prepared in the presence of curcumin yielded the values shown in Table 1.

The resulting curcumin-loaded nanoparticle (NP-CUR) is spherical (Figure 1), and the size obtained was 180.1 ± 19.8 nm, a value slightly lower than that reported by Del Prado-Audelo [20]. Furthermore, the polydispersity index (PDI) obtained was 0.068 ± 0.02, indicating a highly uniform particle population. Finally, the measured zeta potential was −17.05 ± 0.07 mV, indicating that the nanoformulation is highly stable, with low susceptibility to aggregate formation over time.

It is important to note that no apparent difference in particle morphology between empty NPs and NP-CURs was observed by microscopy. However, quantitative measurements reveal that the empty NPs are larger than NP-CUR (224.53 ± 1.91 nm vs. 180.1 ± 19.8, respectively) (Table 1). Another readily apparent difference is that empty NPs are white, while NP-CUR exhibit an intense yellow coloration, consistent with the incorporation of curcumin. This visible indicator suggests that curcumin within the NPs remains unoxidized until administration to the cysticerci, as oxidation leads to loss of the yellow color.

In cysticercus samples, both exposed to curcumin at 1 and 120 h, it was possible to detect curcumin contained in the nanoparticles (NP-CUR) by their ability to fluoresce, indicating first its presence in the cysticerci (Figure 2, merge image) and second that its incorporation into PCL/F68 NP protects it from oxidation until at least 5 days later.

### 3.2. NP-CUR Has a Dose- and Time-Dependent Lethal Effect on T. crassiceps Cysticerci

After quantitation of curcumin concentration in the NP solution was determined (Supplementary Figure S1), *T. crassiceps* cysticerci were exposed to different concentrations for 24, 48, 72, 96, or 120 h. Both untreated cysticerci (control) and those incubated with empty NP remain viable for 120 h (Figure 3A). In contrast, viability decreased over time in all NP-CUR treatments after 48 h. At 120 h, in the presence of 10 µM curcumin, 100% lethality of cysticerci was observed.

Once the treatment condition that produced the highest mortality was determined, the median lethal dose (LD_50_) of NP-CUR on the cysticercus was calculated. For this purpose, the exposure time was set to 120 h, and viability was quantified at each of the concentrations used (Figure 3B). From these values, an LD_50_ of 1.4 µM was determined.

### 3.3. NP-CUR Does Not Consistently Inhibit TGR Activity in Cysticerci

To assess whether the lethal effect of NP-CUR is associated with TGR inhibition, assays were performed to determine TGR activity in cysticerci treated with 2, 5, and 10 µM NP-CUR and compared with that of untreated cysticerci or cysticerci exposed to empty NPs. Basal TGR activity in untreated cysticerci was normalized to 100%. No NP condition significantly altered reductase activity within the first 24 or 48 h, as shown in Figure 4A,B. At 72 h, a significant difference in TGR activity was found only between cysticercus exposed to 5 versus 10 μM NP-CUR (*p* = 0.028) (Figure 4C). On the other hand, at 96 h, a significative decrease in activity was observed only in cysticerci exposed to empty NP with respect to the control (*p* = 0.017) (Figure 4D), whereas at 120 h there was no difference between the control and NP-CUR treatment groups (Figure 4E). Given the lack of consistent concentration- or time-dependent reduction in TGR activity, these isolated significant changes do not indicate a major or consistent inhibition of TGR by NP-CUR. This pattern contrasts with our previous study of free curcumin, where TGR activity consistently decreased with increasing curcumin concentrations [11]. Together these findings suggest that, under the conditions tested, the antiparasitic activity of NP-CUR is not associated with the consistent TGR inhibition previously reported for free curcumin.

### 3.4. Quantification of Reactive Oxygen Species (ROS) in Treated Cysticerci

ROS production in cysticerci was measured following exposure to 2, 5, or 10 µM NP-CUR or empty NPs at the timepoints indicated in Figure 5. ROS levels in untreated cysticerci were normalized to 100%. At 24 and 48 h, a slight but non-significant increase in ROS production was observed in cysticerci treated with both empty NPs and NP-CURs compared to the control group, as shown in Figure 5A,B. At 72 h, a significant increase in ROS was observed in cysticercus exposed to 5 and 10 μM NP-CUR when compared to the empty NP condition (*p* = 0.014 and *p* = 0.010, respectively) (Figure 5C). At 96 h, a significative decrease in ROS was observed in cysticerci exposed to empty NP and 5μM NP-CUR, with respect to control (*p* = 0.028 and *p* = 0.019, respectively) (Figure 5D), whereas at 120 h there was no difference between the control and NP-CUR treatment groups (Figure 5E).

Overall, NP-CUR treatment did not produce a consistent or concentration-dependent increase in ROS over the course of the experiment. The early increase observed at 24 h in all treatment groups, including empty NPs, suggests that nanoparticle exposure itself may contribute to the initial ROS response. Moreover, the absence of a sustained or dose-dependent ROS increase contrasts with the previously reported response following treatment with free curcumin, where increasing curcumin concentrations produced a consistent increase in ROS [11]. These findings suggest that the lethal effect of NP-CUR is not associated with the sustained ROS response previously observed for free curcumin.

### 3.5. NP-CUR Interferes with the Integrity of the Nucleolus of Cysticerci

The effect on the cysticercus structure observed by TEM shows no apparent damage (Supplementary Figure S2A,B) when compared to the damage produced in the tegument and cell parenchyma upon administration of free curcumin (Supplementary Figure S2C,D). In general, the cysticercus structure is maintained after NP-CUR incubation. Importantly, both free curcumin and NP-CUR treatments are compared when cysticerci mortality was 100%, although curcumin concentration and incubation times varied significantly between the two treatments: free curcumin was administered at a concentration 50 times higher (500 μM) than NP-CUR, and the exposure time was only 2 h versus 120 h.

Curiously, ultrastructure differences were found at the nuclear level in cysticerci exposed to NP-CUR, specifically in the nucleoli. Nucleolar bodies were observed as intact, absent, or fragmented as shown in Figure 6A–C. While these three states were present in cysticerci treated with the empty NP or not treated (control), NP-CUR treatment significantly increased the frequency of observed nucleolar fragmentation and loss (*p*-value = 0.002). On the other hand, no significant change (*p*-value = 0.366) was observed when comparing the control to empty NPs, which strongly suggests that the observed effect on the nucleoli structure is related to curcumin treatment and not to the NP delivery method (Figure 6D).

In contrast to the alterations in the nucleus of cysticerci treated with NP-CUR, in the presence of free curcumin, nucleolar structure is maintained and the nucleolus retains its integrity, even at 250 μM free curcumin (Supplementary Figure S3).

## 4. Discussion

The need for antiparasitic plant-based products has increased significantly due to resistance to synthetic drugs currently used [28,29,30]. Cysticercosis remains a major public health problem, and curcumin has shown promising results in the elimination of cysticerci [11]. However, in aqueous media, curcumin undergoes rapid autooxidation, as reported by Schneider and colleagues [13,14]. Oxidation generates transient intermediate molecules that are likely responsible for the antiparasitic activity of curcumin [31]. Indeed, our previous findings uncover a molecular mechanism revealing that curcumin-derived oxidation products (COPs) can inhibit TGR purified to homogeneity [10].

The unstable nature leading to autooxidation of curcumin may mean, however, that the intermediate molecules would degrade before physically reaching parasites in hosts. The next logical step is therefore to improve delivery methods of curcumin that could provide more controlled drug delivery and greater protection against degradation. In this study, we found that nanoformulation enabled curcumin to exert its antiparasitic effect under in vitro conditions at substantially lower concentrations than those required for free curcumin.

It was possible to detect the NP-CUR associated with the cysticercus’s tegument from 1 h after exposure for up to 120 h later, due to the auto-fluorescence of curcumin, as shown in Figure 2. Since fluorescence itself is an intrinsic characteristic of curcumin’s integrity and since this fluorescence remains when non-incorporated NPs were removed (rinsed samples), our data support the conclusion that curcumin enters the cysticercus inside the nanoparticle and that it is stably bound within the tegument. Subsequently, over time, the nanoparticle releases the curcumin into the cytosol, which would mark the beginning of its degradation as demonstrated by the decrease in fluorescence shown in Figure 2. This finding indicates that the NP protects against curcumin oxidation, in accordance with previous reports [6,32], before it is delivered to the parasite.

With NP-CUR, we observe no damage to the ultrastructure of the cysticercus tegument (Supplementary Figure S2A,B). We hypothesize that in the case of free curcumin, autooxidation begins immediately upon contact with the culture medium, exposing the cysticerci to a large amount of ROS (17.5 times greater than that observed under baseline conditions), which is likely responsible for the observed tegument damage [11]. In contrast, when curcumin-loaded nanoparticles are used, our data suggest that curcumin is effectively protected and not exposed to the aqueous medium, so ROS would not be generated in the medium, and the cysticercus tegument would likely not be directly damaged by this mechanism (Supplementary Figure S2).

Exposure to 10 μM NP-CUR for 120 h resulted in 100% cysticercus mortality (Figure 3). In contrast, while in vitro incubation of *T. crassiceps* cysticerci with free curcumin has a dose- and time-dependent lethal effect; complete mortality requires concentrations as high as 500 μM [11]. This concentration is 50 times higher than that required for equivalent lethality using curcumin loaded in PCL/F68 nanoparticles, as shown in the present study by the lower LD_50_ (Figure 3B). Enhanced biological activity of nanoformulated curcumin has been reported in other experimental systems [16,33]. Ghosh et al. reported that curcumin formulated in phospholipid nanodisks produced greater growth inhibition in HepG2 cells and induced apoptosis more readily in a lymphoma culture system than free curcumin [16]. Likewise, Ji et al. reported that curcumin encapsulated in apoferritin nanocages (Cur@HFn) led to greater cellular uptake, cytotoxicity, and apoptosis compared to free curcumin in breast cancer cell lines [33].

The mechanisms responsible for this enhanced biological activity, however, may be sensitive to the nanoformulation or biological context. Ji et al. report increased intracellular ROS in response to Cur@HFn treatment compared to free curcumin, while the greater cysticidal activity of NP-CUR in the present study is accompanied by only a modest increase in ROS (~20%, Figure 5) compared to substantial increases in ROS upon treatment with free curcumin (~100%). Furthermore, TGR activity was not inhibited by NP-CUR (Figure 4).

The greater ROS production observed with free curcumin may, at least in part, reflect the chemical instability of the compound and the formation of modified or degradation products. Our previous findings [10], supported by those of others [12], demonstrate that curcumin undergoes chemical modification after only 10 min of incubation. In contrast, in this study, fluorescence imaging reveals that curcumin remains stable in nanoparticles, even after five days of incubation (Figure 2). Likewise, ROS production following NP-CUR treatment was not strongly dependent on concentration or timepoint (Figure 5). Together, these data suggest that stabilization of curcumin within the nanoparticles may alter the temporal exposure of cysticerci to curcumin and its degradation products, contributing to the lower ROS production observed with NP-CUR compared to free curcumin.

The absence of TGR inhibition or a pronounced ROS response does not exclude effects on other components of the parasite redox system. For example, Luis et al. demostrated that spiroepoxide (an intermediate of curcumin oxidation) can generate in vivo and in vitro adducts with low-molecular-weight thiols, including GSH [14], which in turn would reduce the electron flux to glutathione-dependant peroxidases and impair ROS scavenging, despite residual TGR activity. Our nucleolus imaging analysis (Figure 6) provides an alternative possibility for cysticercus cell death after treatment with NP-CUR that could be independent of parasite cellular redox activity. To further quantify differences in nucleolar ultrastructure, in each TEM image, we measured the normalized grayscale intensity along a line drawn across the nucleus through the nucleolus or the corresponding region when the nucleolus was absent or fragmented (Figure 6E). The intensity profiles differed significantly between NP-CUR-treated and untreated control cysticerci, while no significant difference was found between control and empty NP cysticerci.

In addition to the well-documented role of the nucleolus in ribosome biogenesis [34,35], it is now recognized for its participation in genome organization [36], epigenetic regulation [37,38], and as a sensor and coordinator of cellular stress responses [39]. These functions are particularly relevant in the context of curcumin, which has been reported to affect chromatin organization and epigenetic regulation in several biological systems. In support of curcumin’s role in chromatin remodeling, human hepatoma cells exposed to curcumin exhibited significant decreases in histone acetylation, a histone acetyltransferase, and a histone deacetylase [40,41,42]. Curcumin has been shown to induce epigenetic changes that affect gene expression in *Trypanosoma cruzi* [32] and in rats [40]. Likewise, curcumin has been directly associated with alterations in nucleolar organization. In a particularly relevant study using *T. cruzi* as a study model, Bortolami et al. [38] demonstrate that curcumin (5 and 10 μM) produces nucleolus disorganization, similar to the alterations shown in Figure 6. These findings provide a precedent for an association between curcumin exposure and changes in nucleolar architecture in a parasitic organism.

The nucleolar fragmentation observed in NP-CUR-treated cysticerci suggests that curcumin may disrupt nucleolar function, a process known to activate apoptotic signaling pathways. Indeed, curcumin has been reported to induce apoptosis in several systems [43,44,45,46,47]. In adult worms of *S. mansoni*, for example, curcumin reduces parasite viability and induces apoptotic damage, including DNA fragmentation [44]. Apoptosis is frequently mediated by activation of the p53 pathway [48]; the tumor suppressor protein p14^arf^ stabilizes p53 through MDM2, thus preventing apoptosis [49]. In SF-767 glioma cells, in the presence of 20 μM curcumin indicated that after 8 h, p14^arf^ shifts to the nucleoplasm and p53 increases levels, leading to apoptosis [47]. In addition, these authors demonstrate that curcumin co-localized with nucleophosmine, a nucleolus marker phosphoprotein. To test the possibility that cysticercus mortality was due to apoptosis, we performed two assays: a DNA fragmentation assay to assess genomic DNA integrity [50] and an assay measuring the release of cytochrome c into the soluble cytosolic fraction following mitochondrial separation by centrifugation [51]. However, our preliminary results showed that neither assay provided evidence for activation of the apoptotic pathway in NP-CUR-treated cysticerci (Supplementary Figure S4).

While we cannot definitively exclude apoptosis as a contributor to NP-CUR-induced cysticerci mortality, our findings suggest that an alternative mechanism may be involved. The nucleolus is an established sensor of cellular stress [52,53], and its structure and function can be altered by diverse stressors, including oxidative stress and nutrient deprivation. Because the nucleolus is also the site of ribosome biogenesis, disruption of this process can trigger nucleolar stress and lead to characteristic changes in nucleolar organization. Similar alterations in nucleolar morphology to those observed in the present study (Figure 6D,E) have been reported following treatment with compounds that disrupt ribosome biogenesis or inhibit cyclin-dependent kinases [54,55]. These observations support the hypothesis that NP-CUR treatment perturbs nucleolar homeostasis. Such perturbation could contribute to cellular stress and, if unresolved, could ultimately lead to cell death.

## 5. Conclusions

In summary, our results demonstrated that exposure to 10 µM NP-CUR for 120 h resulted in 100% mortality of the cysticerci, suggesting that NP-CUR can achieve complete antiparasitic activity at a substantially lower concentration than free curcumin. Our results further indicate that curcumin retains its antiparasitic activity in both its free form and when incorporated into nanoparticles, although the concentration required to achieve complete mortality and the cellular responses associated with treatment differ between the two formulations. In particular, NP-CUR treatment was not associated with a consistent increase in ROS or inhibition of TGR activity, while alterations in nucleolar morphology were observed. These findings suggest that nanoformulation may alter the biological response to curcumin, although the specific mechanisms underlying NP-CUR-induced toxicity remain to be determined. This distinction should be considered in future studies evaluating nanoparticle-based curcumin formulations as potential antiparasitic therapies.

## Figures and Tables

**Figure 1:**
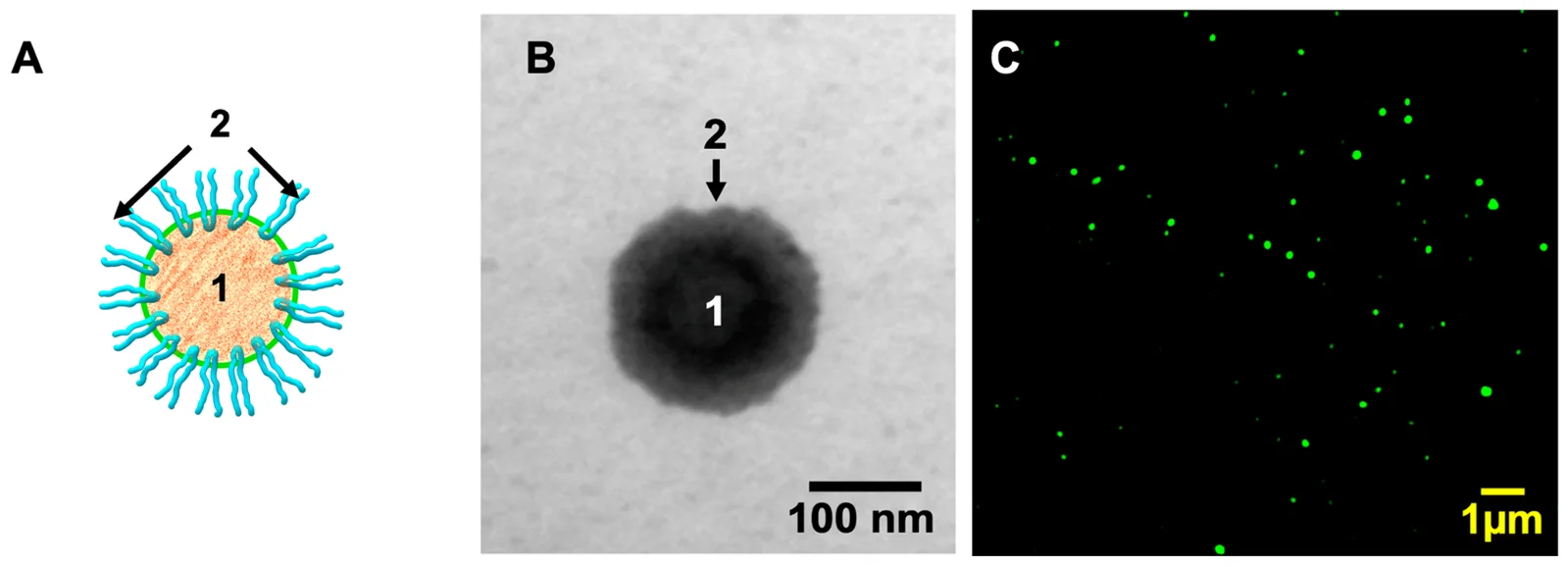
Characterizacion of PCL/F68-CUR NP. (**A**) Schematic representation of PCL/F68 NP, while (**B**) shows an image of a PCL/F68 NP obtained by transmission electron microscopy. Both images show the hydrophobic polycaprolactone core (1) as well as the hydrophilic regions of Pluronic© F-68 exposed to the solvent (2); (**C**) shows the fluorescence of curcumin-loaded NPs (NP-CUR). The image was obtained by confocal fluorescence microscopy (excitation 488 nm/emission 520–580 nm).

**Figure 2:**
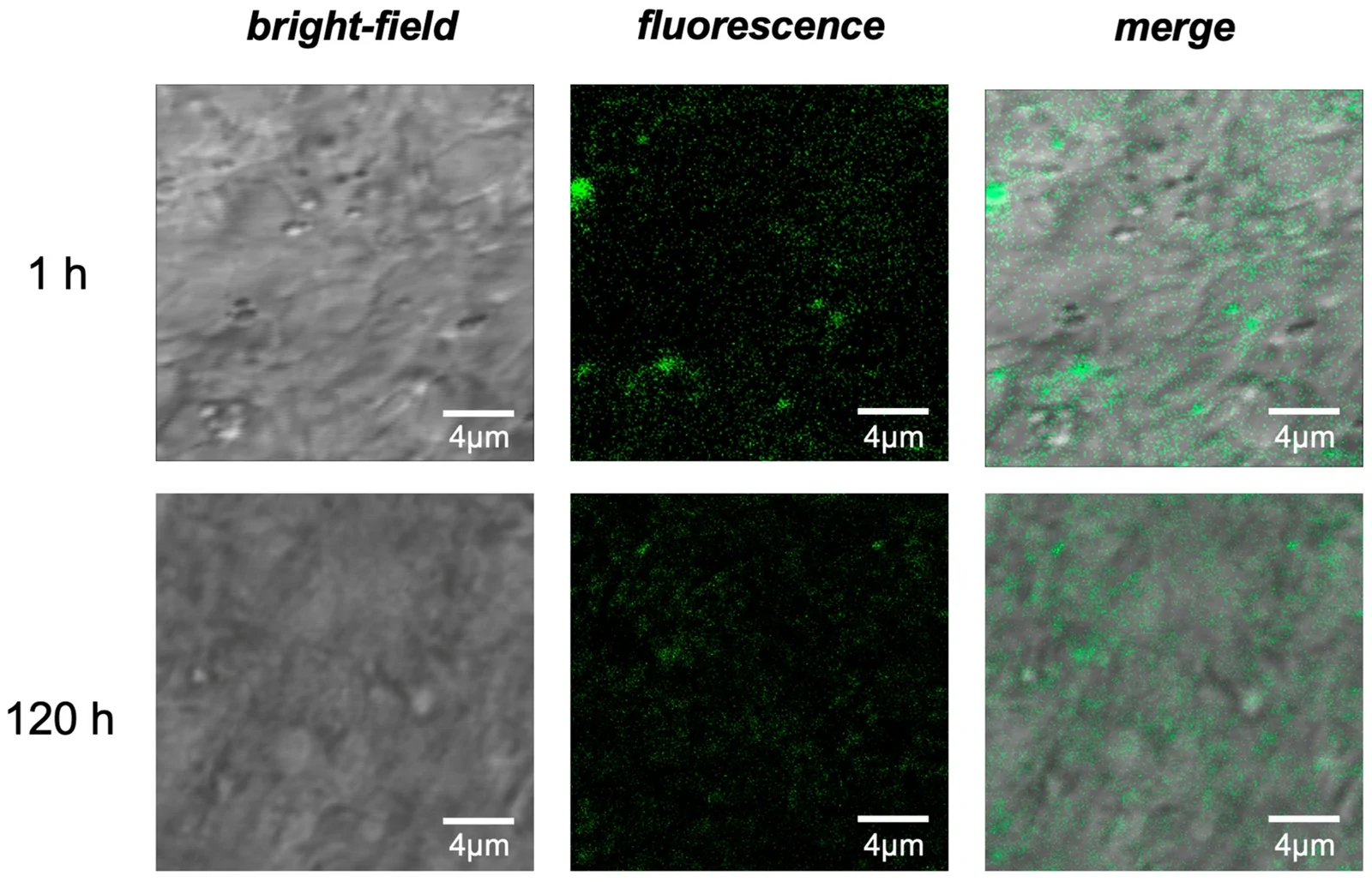
The PCL/F68-CUR NPs are associated with the inside of the cysticercus’s tegument. Cysticercus exposed to NP-CUR and observed using bright-field microscopy and confocal fluorescence microscopy (excitation 488 nm/emission 520–580 nm) are shown. (**Top row**) after one hour of incubation; (**bottom row**) after 120 h of incubation. Scale bar, 4 µm. The images shown are representative of three independent experiments.

**Figure 3:**
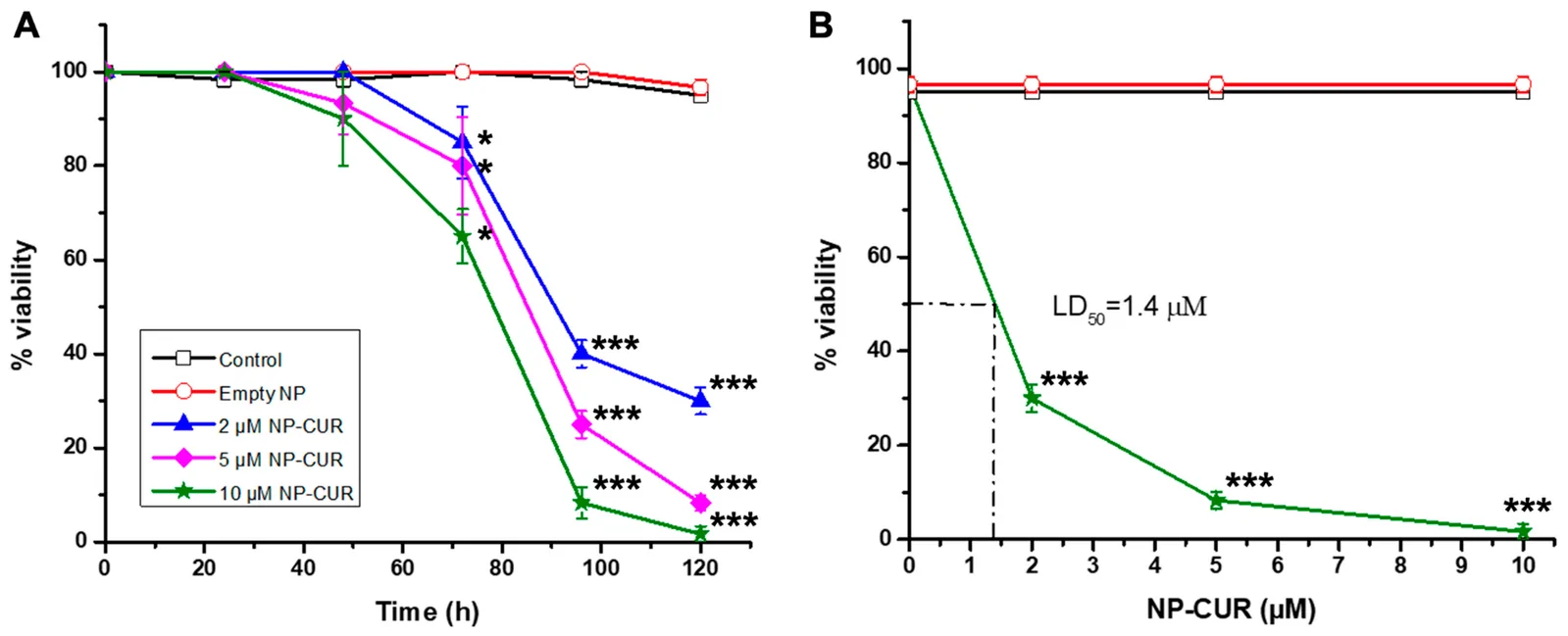
NP-CUR has a lethal effect on *T. crassiceps* cysticerci. (**A**) The effect of curcumin-loaded nanoparticles on cysticercus viability is shown. *C*urcumin concentrations during incubation were 2 (blue triangles), 5 (magenta diamonds), and 10 µM (green stars), respectively. As a control, the viability of cysticerci without treatment (open squares) and cysticerci exposed to empty NPs (open circles) was quantified over time. (**B**) The effect of the curcumin concentration contained in the NPs at a fixed time (120 h) is shown. The results are shown as the mean of three independent experiments ± SEM. * *p* < 0.05; *** *p* < 0.001.

**Figure 4:**
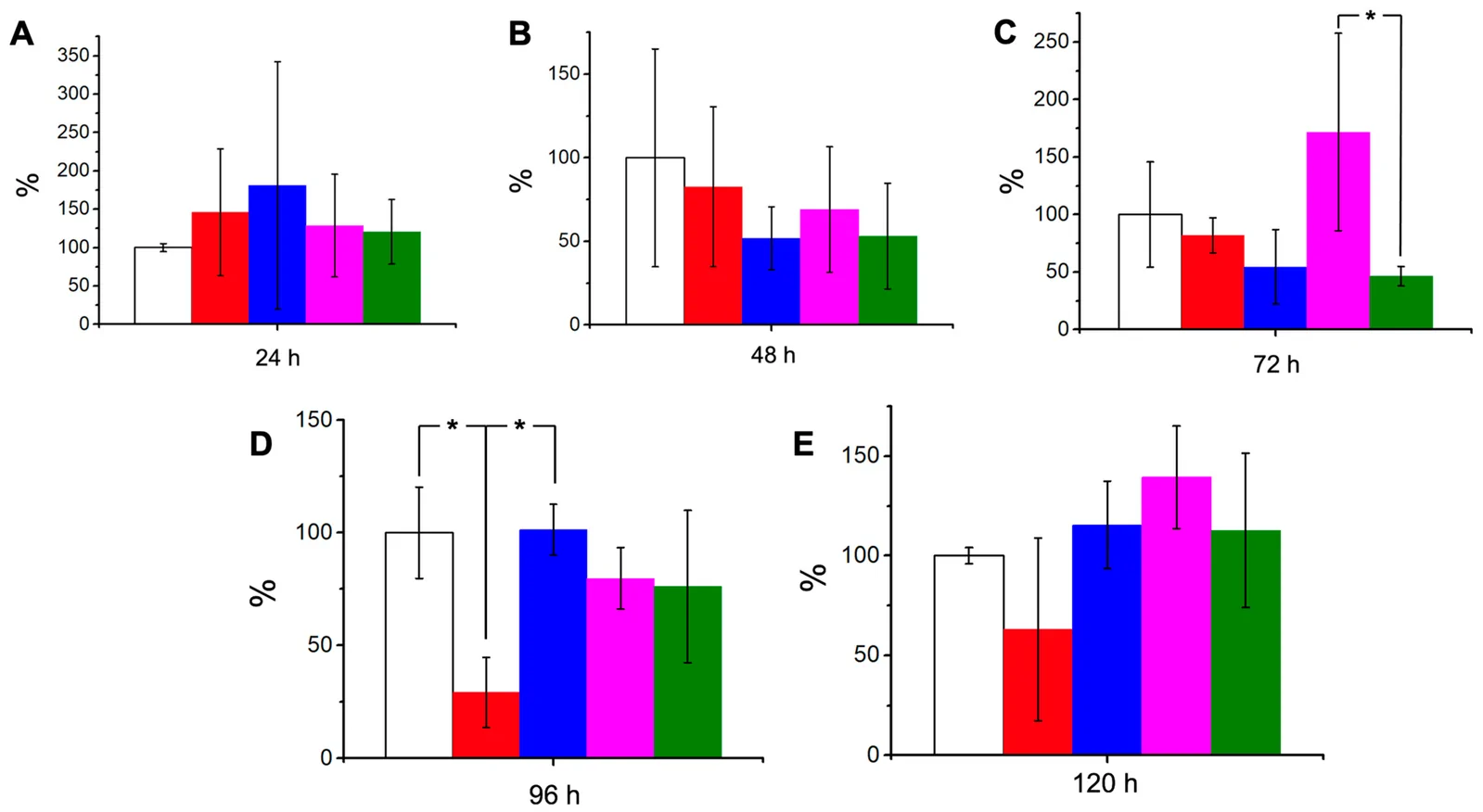
CUR- NPs do not significantly modify TGR activity on *T. crassiceps* cysticerci. The effect of curcumin-loaded nanoparticles on TGR activity at (**A**) 24, (**B**) 48, (**C**) 72, (**D**) 96, and (**E**) 120 h is shown. Data are presented as a percentage relative to control, which is set to 100%. Curcumin final concentrations during incubation are shown with the following colors: 2 (blue), 5 (magenta), and 10 µM (green), respectively. As a control, the TGR activity of cysticerci without any treatment (white) and cysts exposed to empty NPs (red) is shown. The results are shown as the mean of three independent experiments ± SD. * *p* < 0.05.

**Figure 5:**
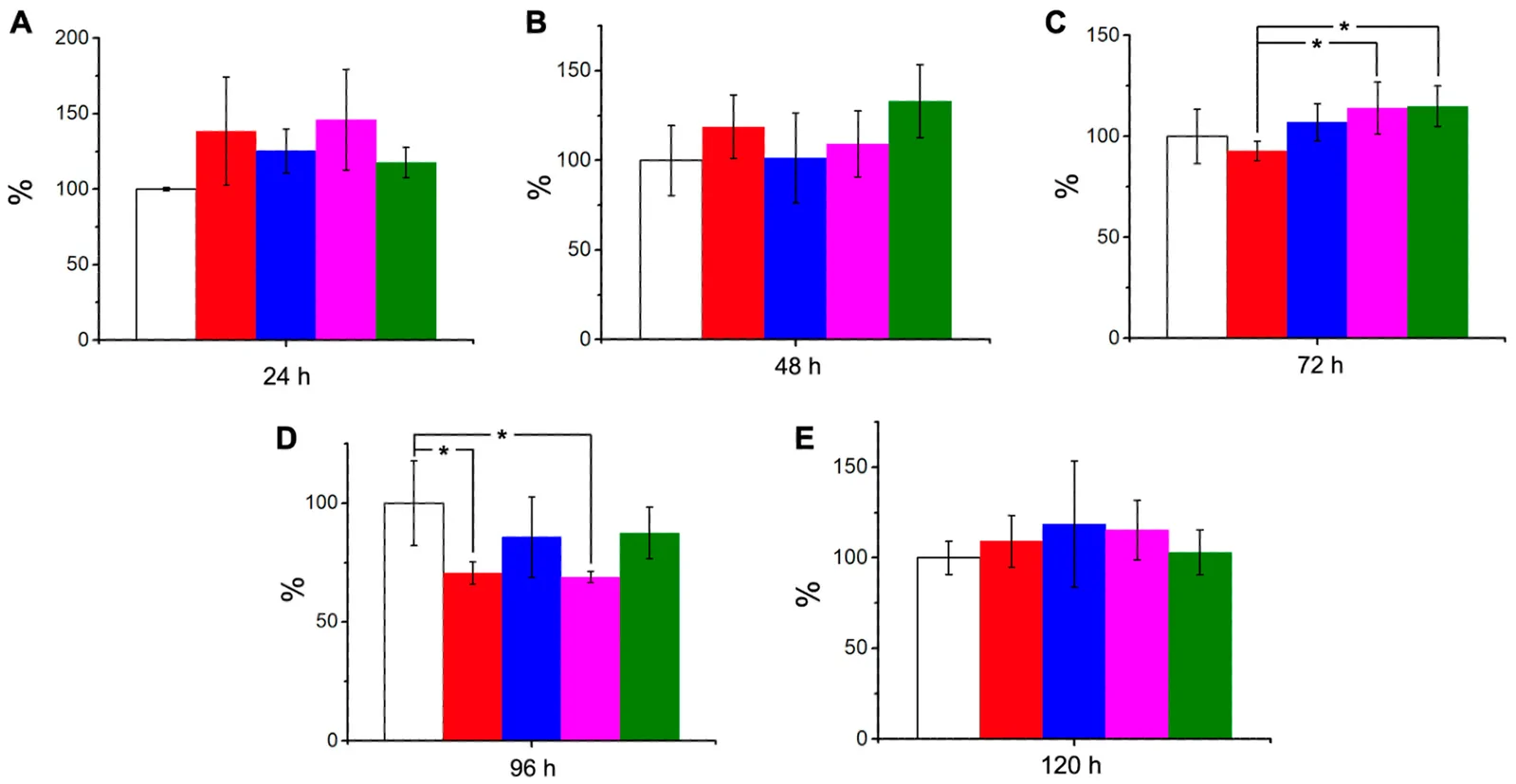
Quantification of ROS generation in cysticerci exposed to NPs and NP-CUR. ROS generation in whole cysticerci at (**A**) 24, (**B**) 48, (**C**) 72, (**D**) 96, and (**E**) 120 h is shown. Data are presented as a percentage relative to control, which is set to 100%. Curcumin final concentrations during incubation are depicted with the following colors: 2 (blue), 5 (magenta), and 10 µM (green). As a control, ROS production of cysticerci without any treatment (white) and cysticerci exposed to empty NPs (red) is shown. The results are shown as the mean of three independent experiments ± SD. * *p* < 0.05.

**Figure 6:**
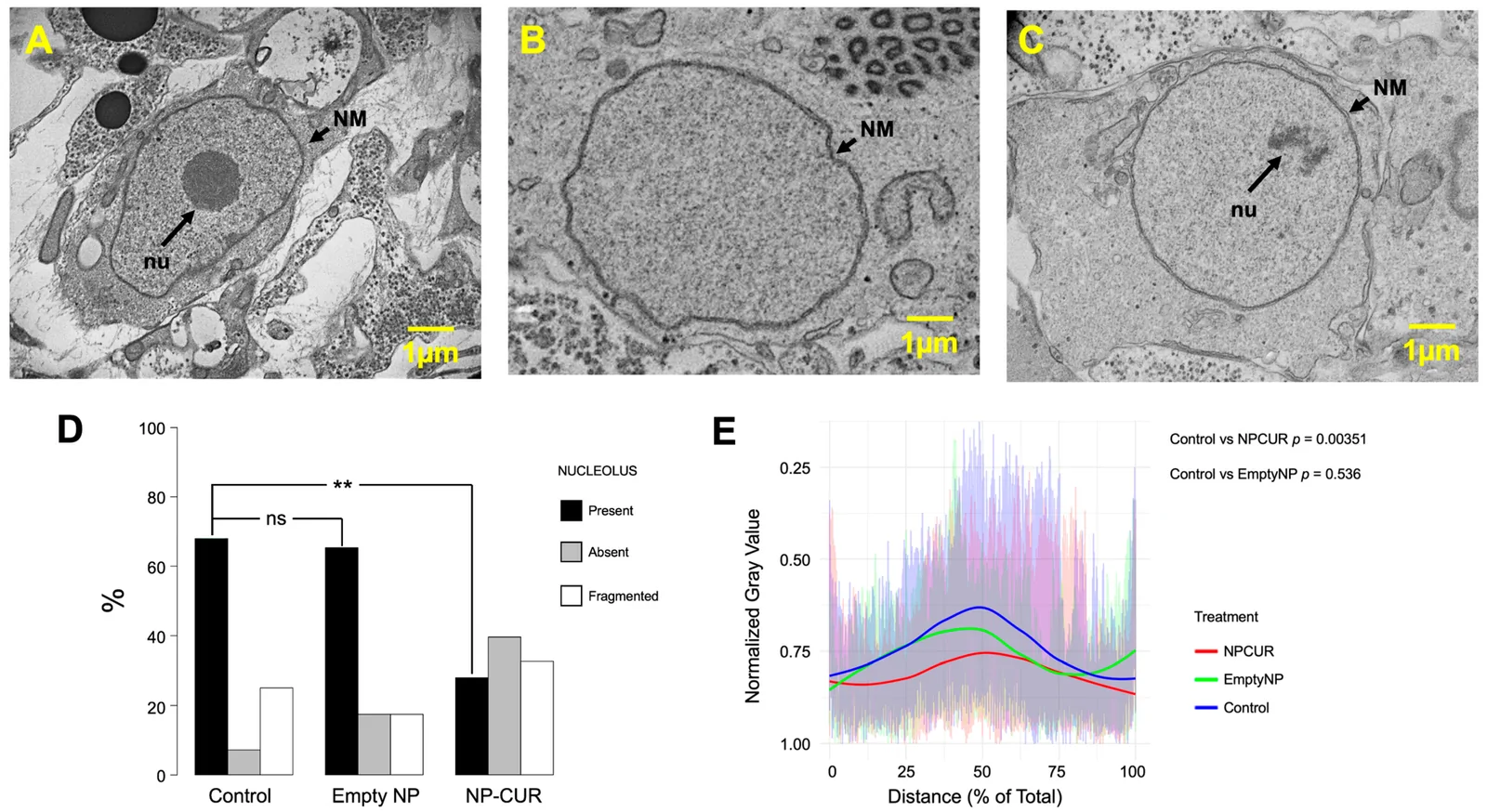
NP-CUR induces alterations in the nucleus. TEM analysis of cysticerci nuclei incubated for 120 h shows examples of nucleoli that are present (**A**), absent (**B**), or fragmented (**C**). (**D**) shows the proportion of the three types of nucleoli found in untreated cysticerci as well as in cysticerci treated with empty NP or NP-CUR (10 μM), represented as % total for each condition. Significance was determined by multinomial mixed-effects logistic regression model. ns, not significant. *p* > 0.05 (*p*-value = 0.36659); **, *p* < 0.01 (*p*-value = 0.00269). (**E**) Normalized Gray value obtained from the analysis of TEM images plotted by distance across the nucleus. Individual intensity profiles are shown as faint lines, and solid lines represent LOESS-smoothed curves for each treatment, control (blue), empty NP (green) and NP-Cur (red). Statistics, Fisher’s Z test: control vs. NP-CUR, **, *p* = 0.003; control vs. empty NP, ns, *p* = 0.536. Abbreviations: nu, nucleolus; NM, nuclear membrane.

**Table 1:** Physicochemical characterization of PCL/F68 NP. The parameters were determined using four independent NP samples and are presented as mean ± SD.

Parameter	PCL/F68 (Empty NP)	PCL/F68-CUR (Curcumin-Loaded NP)
Size (nm)	224.53 ± 1.91	180.1 ± 19.8
PDI	0.028 ± 0.027	0.068 ± 0.02
Zeta potential (mV)	−22.43 ± 0.40	−17.05 ± 0.07

## Data Availability

The original contributions presented in this study are included in the article and Supplementary Materials. The raw data supporting the conclusions of this article will be made available by the authors on request.

## Supplementary Materials

The following supporting information can be downloaded at https://www.mdpi.com/article/10.3390/antiox15101226/s1. Figure S1. Absorption spectrum of PCL/F68 NPs. (A) The absorption spectrum of empty NPs is shown. This absorption is due exclusively to PCL and Poloxamer 188 and was subtracted from the corresponding measurements. (B) The absorption spectrum of a representative sample of the NP suspension loaded with curcumin, corresponding to 5.4 µM of curcumin, is shown. *INSERT*. Standard curve shows the correspondence between the curcumin concentration and its absorption at 426 nm; Figure S2. Comparative TEM of cysticerci exposed to free curcumin and curcumin-loaded NPs. 10 μM NP-CUR (120 h of exposure) shows no damage to tegument (A,B), while 500 μM free curcumin (2 h of exposure) damages cysticerci tegument (C,D). Abbreviations: BM, basal membrane; m, muscle; Mt, microtriches. The asterisk indicates the region corresponding to the tegument; Figure S3. Free curcumin does not induce alterations in the nucleus. Nuclei TEM analysis of cysticerci incubated for 2 h with free curcumin in culture medium. (A) Control; (B) vehicle control (DMSO 5%); and (C) 50, (D) 250, or (E) 500 µM of free curcumin. Abbreviations: nu, nucleolus; Figure S4. NP-Cur does not induce apoptosis. Two late markers of apoptosis were evaluated in cysticerci incubated in the presence of NPs for 120 h. (A) DNA fragmentation. Genomic DNA was extracted from 40 mg samples of cysticerci—(Line 1) control, (Line 2) exposed to empty nanoparticles, and (Line 3) exposed to curcumin-loaded nanoparticles (10 µM)—using the Animal Genomic DNA Miniprep Kit (BioBasic BS427, Canada, CAD) and following the manufacturer’s instructions. The DNA obtained was loaded onto and visualized by electrophoresis in 1.5% agarose gels in the presence of GelRed^®^ (GelRed Nucleic Acid Stain, Biotium); Line 4 shows the molecular weight markers. (B) Release of cytochrome c into the cytosol. The cytosolic fraction was obtained from cysticercus exposed to nanoparticles loaded with curcumin (10 µM) for 120 h, using differential centrifugation. An aliquot of this fraction was analyzed using spectrophotometric scanning. In (CyC) shows the absorption spectrum of horse heart cytochrome c, while (sample) shows the absorption spectrum of the aliquot from the cysticerci. The images shown are representative of two independent experiments.

## Abbreviations

The following abbreviations are used in this manuscript:

CICUAL	Institutional Animal Care and Use Internal Committee
C-LNCs	Curcumin-loaded lipid-core nanocapsules
CM-H_2_DCFDA	5-(6-)chloromethyl-2′,7′-dichlorodihydrofluorescein diacetate
CUR	Curcumin
DMSO	Dimethylsulfoxide
GSSG	Oxidized glutathione
LD_50_	Half lethal doses
MWCO	Molecular weight cut-off
NADPH	Reduced nicotinamide-adenin dinucleotide
NPs	Nanoparticles
NP-CUR	Nanoparticles loaded with curcumin
PBS	Phosphate-buffered saline
PCL/F68	Polycaprolactone/Pluronic-F68 nanoparticles
PDI	Polydispersity Index
PMSF	Phenylmethylsulfonyl fluoride
ROS	Reactive oxygen species
SEM	Standard error of mean
TEM	Transmission electron microscope
TGR	Thioredoxin glutathione reductase
