# Supplementary material for: Nanoencapsulation Transforms Curcumin’s Antiparasitic Activity: Reduced Dose and Altered Mechanism in Taenia crassiceps Cysticerci
Source: Antioxidants (Basel). 2026 Sep 23;15(10):1226. doi: 10.3390/antiox15101226 (PMC13700812; doi:10.3390/antiox15101226)
Supplement: Supplementary file 1 [file antioxidants-15-01226-s001.zip › antioxidants-4520310-supplementary.pdf]

Suplementary materials

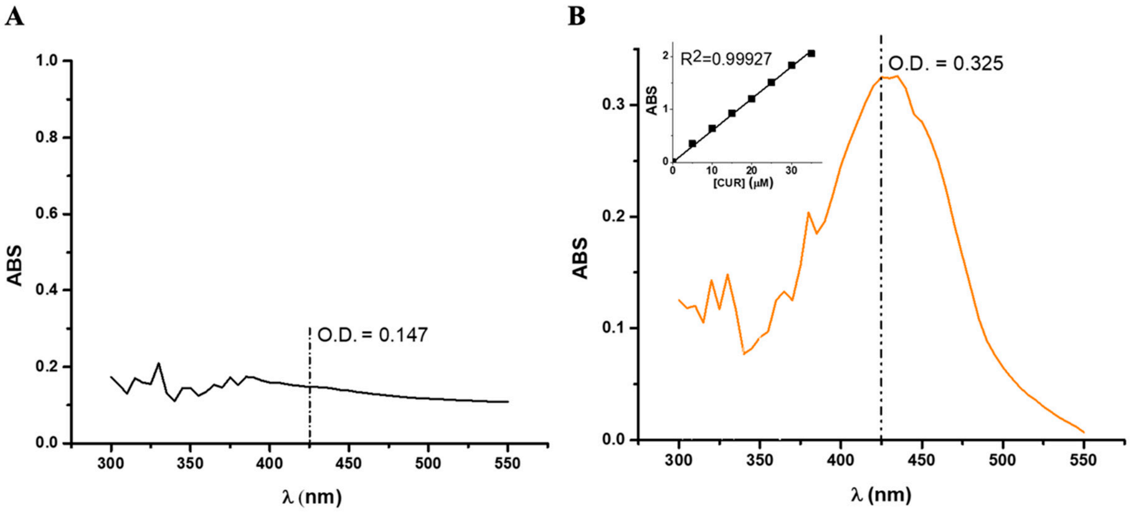

Figure S1. Absorption spectrum of PCL/F68 NPs. (**A**) The absorption spectrum of empty NPs is shown. This absorption is due exclusively to PCL and Poloxamer 188 and was subtracted from the corresponding measurements. (**B**) The absorption spectrum of a representative sample of the NP suspension loaded with curcumin, corresponding to 5.4 μM of curcumin, is shown. *INSERT*. Standard curve shows the correspondence between the curcumin concentration and its absorption at 426 nm.

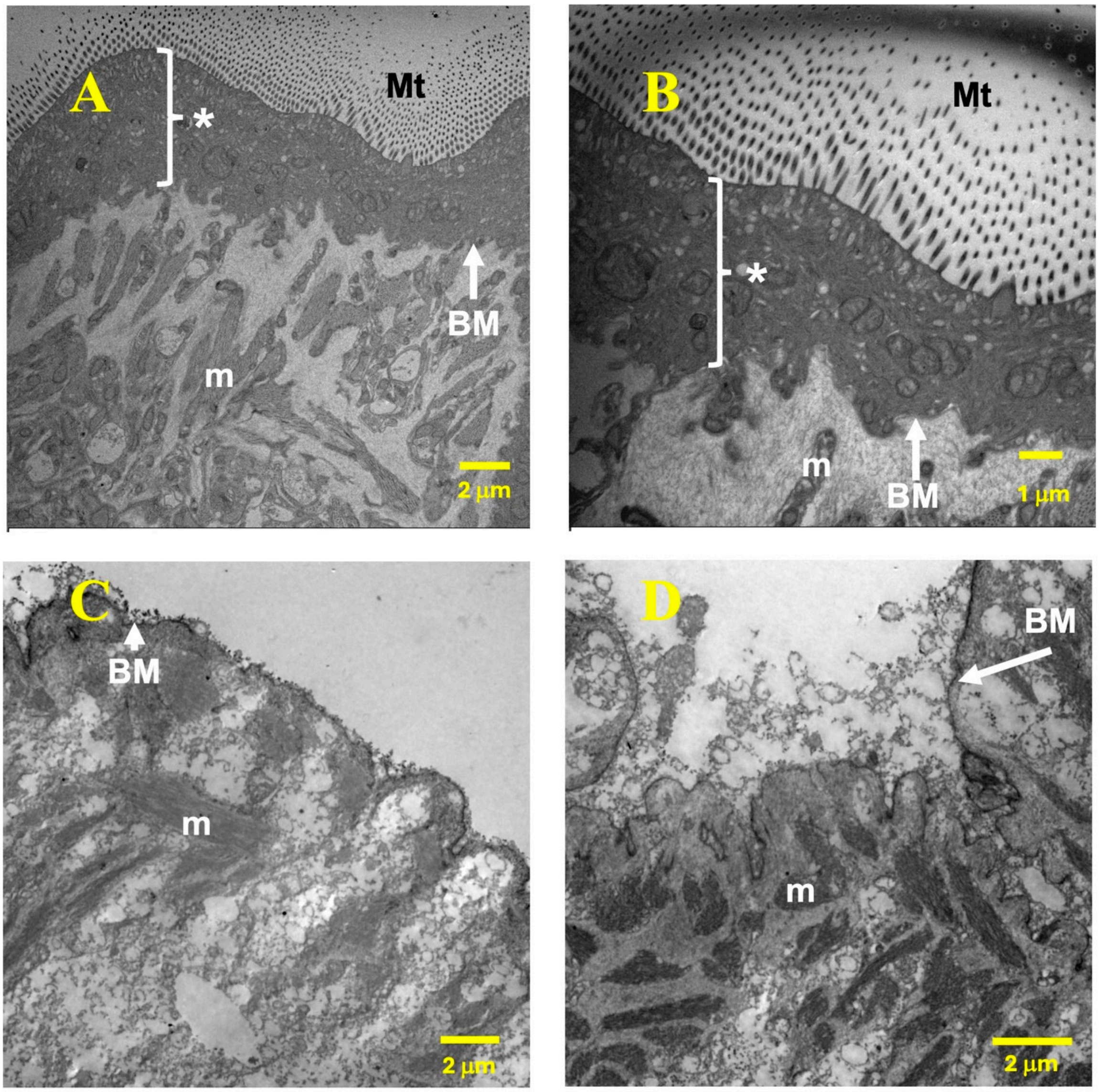

Figure S2. Comparative TEM of cysticerci exposed to free-curcumin and curcumin-loaded NPs. 10 µM NP-CUR (120 h of exposure) shows no damage to tegument (**A, B**), while 500 µM free-curcumin (2 h of exposure) damages cysticerci tegument (**C, D**). Abbreviations: BM, basal membrane; m, muscle; Mt, microtriches. The asterisk indicates the region corresponding to the tegument.

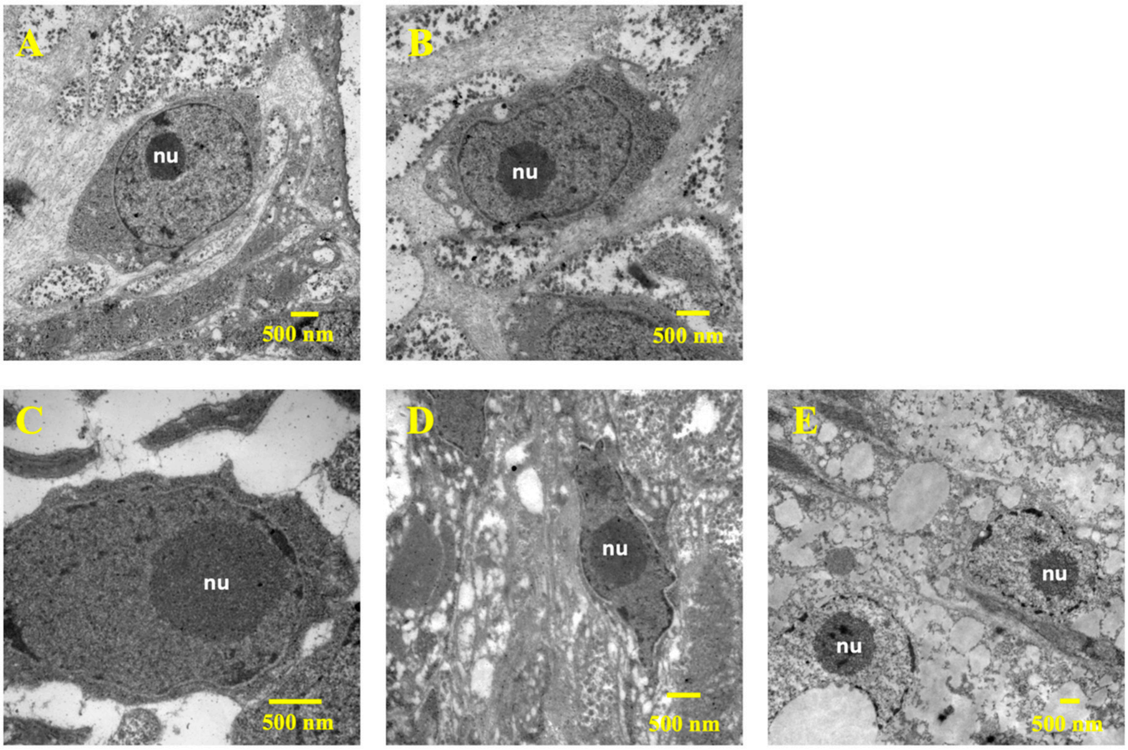

Figure S3. Free-curcumin does not induce alterations in the nucleus. Nuclei TEM analysis of cysticerci incubated during 2 h with free curcumin in culture medium. (**A**) control; (**B**) Vehicle control (DMSO 5%); and (**C**) 50, (**D**) 250, or (**E**) 500 μM of free-curcumin. Abbreviations: nu, nucleolus.

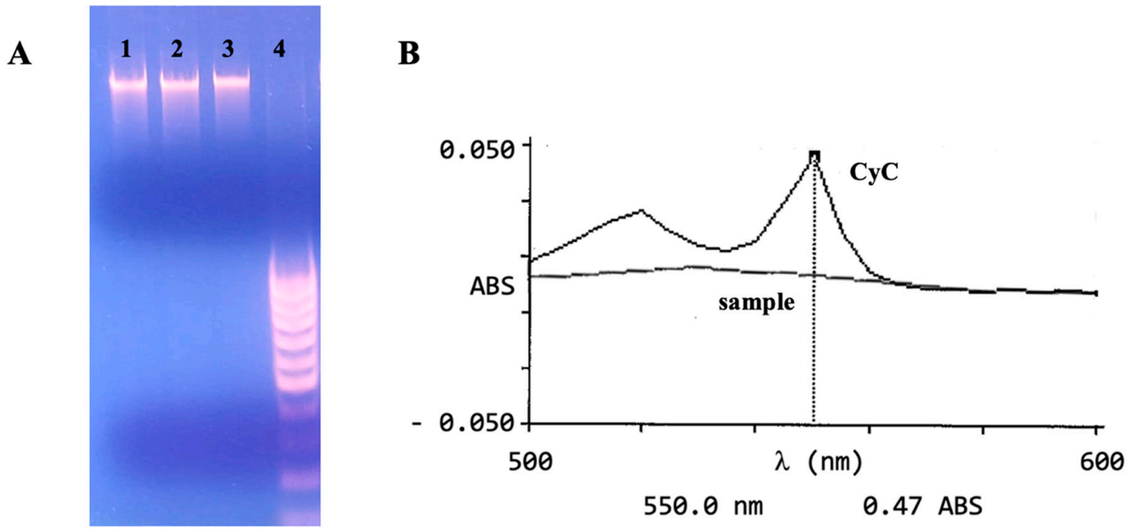

Figure S4. NP-Cur does not induce apoptosis. Two late markers of apoptosis were evaluated in cysticerci incubated in the presence of NPs for 120 hours. (**A**) *DNA fragmentation*. Genomic DNA was extracted from 40 mg samples of cysticerci— (*Line 1*) control, (*Line 2*) exposed to empty nanoparticles, and (*Line 3*) exposed to curcumin-loaded nanoparticles (10 μM)—using the Animal Genomic DNA Miniprep Kit (BioBasic BS427, Canada, CAD) and following the manufacturer's instructions. The DNA obtained was loaded onto and visualized by electrophoresis in 1.5% agarose gels in the presence of GelRed® (GelRed Nucleic Acid Stain, Biotium); *Line 4* shows the molecular weight markers. (**B**) *Release of cytochrome c into the cytosol.* The cytosolic fraction was obtained from cysticercus exposed to nanoparticles loaded with curcumin (10 μM) for 120 h, using differential centrifugation. An aliquot of this fraction was analyzed using spectrophotometric scanning. In (CyC) shows the absorption spectrum of horse heart cytochrome *c*, while (sample) shows the absorption spectrum of the aliquot from the cysticerci. The images shown are representative of two independent experiments.
